# Preparation, Characterization, and Application of Modified Starch/Chitosan/Sweet Orange Oil Microcapsules

**DOI:** 10.3390/foods11152306

**Published:** 2022-08-02

**Authors:** Liang Qiu, Hui Ma, Qinghua Luo, Chan Bai, Guangquan Xiong, Shiwei Jin, Juguang Wang, Xiaoyan Zu, Hailan Li, Tao Liao

**Affiliations:** 1 Hubei Engineering Research Center for Agricultural Products Irradiation, Institute of Agro-Products Processing and Nuclear Agricultural Technology, Hubei Academy of Agricultural Sciences, Wuhan 430064, China; qiuliang@hbas.com (L.Q.); baichan09@hbas.com (C.B.); xiongguangquan@hbaas.com (G.X.); juguangw@hbas.com (J.W.); zuxiaoyan@hbaas.com (X.Z.); hl.li@hbaas.com (H.L.); 2 Key Laboratory of Cold Chain Logistics Technology for Agro-Product, Ministry of Agriculture and Rural Affairs, Wuhan 430064, China; 3 Key Laboratory of Catalysis and Energy Materials Chemistry of Education, South-Central University for Nationalities, Wuhan 430074, China; 2020120311@scuec.edu.cn (H.M.); luoqinghua@scuec.edu.cn (Q.L.); jinsw@mail.scuec.edu.cn (S.J.)

**Keywords:** sweet orange essential oil, antibacterial activity, porous starch, microcapsule, crawfish

## Abstract

Aquatic products have an important role in global agriculture, but the challenges associated with preservation have limited their marketability. Essential oil (EO), such as sweet orange oil (SOEO), has been widely used for preservation due to its excellent antibacterial ability. However, the volatilization of EO limits its application in food preservation. In this study, SOEO was extracted from sweet orange peel by steam distillation and then stored in microcapsules. The components of the microcapsules were as follows: the porous starch was chosen as an adsorbed substrate to store SOEO (PS/SOEO), and sodium alginate (SA) and chitosan (CMCS) were used as shell material to delay the volatilization of SOEO using the sharp pore coagulation method. Our results showed that the main antibacterial ingredients in SOEO were aldehydes (33.93%) and d-limonene (15.38%). The microcapsules were of an irregular shape (oval), and the size of the microcapsules was 1.2 ± 0.1 cm as measured by a digital micrometer. Scanning electron microscopy (SEM) results showed that there were a lot of pores on the surface of the starch after modification, but sodium alginate and chitosan could well encapsulate these pores. The results of Fourier transform infrared (FTIR) spectroscopy and X-ray diffraction (XRD) analysis also showed that SOEO was successful encapsulated into the porous starch. The results of compression test and releasing kinetics studies suggested that CMCS and SA improved the mechanical and slow-releasing ability of SOEO microcapsules. The best antibacterial performance was obtained when 0.8 g of SOEO microcapsules was added. Finally, the shelf life of crawfish could be extended to 6 days by SOEO microcapsule (1/10 g, SOEO microcapsule/crawfish) under room temperature. These results provide a systematic understanding of the antibacterial capabilities of sweet orange essential oil microcapsules, which can contribute to the development of preservation methods for aquatic products.

## 1. Introduction

Aquaculture is an essential economic pillar industry in many countries. However, due to its high nutrition, high moisture, and low connective tissue content, aquatic products easily decay, leading to severe economic loss. In recent years, plant essential oil (EO) has been considered a potential food preservative because of its antioxidant, biophilic, and antibacterial properties [1,2,3]. It is relatively simple to extract using the leaves, roots, and fruits of plants as raw materials through simple squeezing and distillation [4]. However, the direct use of EO has many limitations, such as lower water solubility, high volatility, high sensitivity to environment, and strong odor. These limitations inhibit its use as a preservation material in food packaging engineering [5]. How to improve the stability of EO has become a key issue for researchers.

At present, microencapsulation technology is widely used in the food, biological, and pharmaceutical fields [6]. Studies have shown that microencapsulation could improve the stability of bioactive compounds and endow EO with sustained-release characteristics that could extend the actuation duration [7]. Besides playing an antibacterial role, EO can also protect or improve the physicochemical properties of food [8]. Sotelo et al. used thyme essential oil as the core material to prepare microcapsules by coprecipitation and found that EO microcapsules had the same antibacterial ability as thyme essential oil [9]. This suggests that microencapsulation can not only effectively prevent the instability of EO and increase its contact area with food but also be easily dispersed in the food surface to inhibit microbial growth and proliferation [10]. Microcapsules can be prepared by interfacial polymerization [11], spray drying [12], sharp pore-coagulation bath [13], etc. Among these methods, pore-coagulation bath has the advantages of simple operation and low cost. Moreover, it can be operated at low temperature, which can reduce loss of EO during the embedding process [14]. Many researchers have used pore-coagulation bath to produce EO microcapsules, such as star anise oil, clove oil, etc. [15]. The components of microcapsules are substrate and shell. The substrate determines the amount of EO in microcapsules, while the shell can enhance the stability of EO and prolong its releasing time. The substrate is mainly produced by adsorption materials as it stores essential oils through adsorption [16]. As a renewable natural polymer material, starch is a natural, cheap, and biodegradable polymer. It is also the main source of carbohydrates in the human diet [17]. Therefore, starch products have been widely used to carry active ingredients, such as antiseptic, antioxidants, colorants, spices, and nutrients, which can be used as a kind of conveying system [18]. Unlike other active ingredients, EO is more sensitive to the environment. The core needs higher mechanical strength and good qualitative property [19]. Therefore, modification is needed for starch to embed EO. According to the structure and physicochemical properties of starch, the main modification methods are physical and chemical [20]. For example, Wang et al. used porous starch that was produced by enzyme as a core of microcapsule to carry clove essential oil and found that the modified microcapsule had strong heat resistance and significant inhibitory effect on the mold spore [21]. Dons et al. found that EO-modified starch microcapsule could minimize the effects on food taste and act as a substance to increase bioactivity [22]. Thus, modified starch is an effective and innovative substrate to maintain the effectiveness of EO.

The shell of microcapsules can separate the substrate from the external environment, reducing the impact of oxygen, heat, lighting, and pH on the EO and enhancing its stability [23]. Many researchers have used chitosan to embed EO microcapsules because of its biodegradability and safety. For example, Sotelo et al. used chitosan to prepare thyme essential oil microcapsule and found that the embedding rate of the microcapsules was more than 68% and had a strong inhibitory effect on *Bacillus cereus* [9].

However, the forming capability of chitosan is very poor, so it needs to be combined with other materials. Many studies have shown that the combined use of two or more shell materials is beneficial to improve the loading capacity and stability of microcapsules [24]. Sodium alginate (SA) is a linear polysaccharide extracted from seaweed and can be cross-linked to chitosan to form hydrogels or insoluble polymers [25]. Han et al. used chitosan and SA as substrates to store thyme essential oil by the layer self-assembly method. The results showed that the embedding rate of microcapsule was up to 80.23% [26].

In the above-described context, the objectives of this study were to (1) prepare a porous corn starch using the enzymolysis method; (2) use porous corn starch as a substance to adsorb sweet orange essential oil and sodium alginate (SA) and modified carboxymethyl chitosan (CMCS) as shell to protect the SOEO; (3) characterize the resulting materials by Fourier transform infrared spectroscopy (FT-IR), pressure resistance analysis, X-ray diffraction (XRD), and scanning electron microscopy (SEM); (4) explore the effective antibacterial ingredients of sweet orange oil and its release kinetics; (5) test the antibacterial properties of SOEO and compare it with natural starch; and (6) determine the preservation of crawfish by SOEO microcapsules.

## 2. Materials and Methods

### 2.1. Materials

The main materials used were natural starch (food grade), ethanol, n-hexane, acetic acid, disodium hydrogen phosphate, citric acid, calcium chloride anhydrous, sodium hydroxide, potassium bromide (KBr), magnesium oxide (MgO), nutrient agar, and carboxymethyl chitosan (CMCS); all were analytically pure materials and purchased from Sinopharm Chemical Reagent Co. (Shanghai, China). Sodium alginate (SA) was purchased from Shanghai Maclean Biochemical Technology Co. (Shanghai, China). BCP (bromocresol purple plate count agar) medium with 0.2% soluble starch was purchased from Beijing Wokai Biotechnology Co. (Beijing, China). Two strains, *Aeromonas sobria* (ATCC43979) and *Shwanella putrefaciens* (ATCCBAA-1097), were purchased from Shanghai Preservation Biotechnology Centre (Shanghai, China). Sweet orange essential oil (SOEO) was sourced from sweet orange essential peel. The chitosan used in this experiment was treated with 10 KGY irradiation (Hubei Irradiation Experimental Center (Beijing, China), ^60^Co-ray; the irradiation dose rate was 6.25 Gy/min, and the irradiation doses were set at 20, 40, 60, 80, and 100 KGy.

### 2.2. Preparation of Modified Porous Starch

According to the method of Fu et al. [27], the orthogonal test was used to find the best conditions of porous modified starch by enzymatic digestion, with the ratio of enzyme to starch, the pH of the buffer solution, and the reaction temperature set as three factors and three levels (Table 1) [28,29]. Natural corn starch was mixed with citric acid/disodium hydrogen phosphate buffer solution at a mass ratio of 1:8, placed in a magnetic heating stirrer, and stirred in a water bath at 25 °C for 0.5 h. Next, α-amylase and glucoamylase were added, and the reaction was terminated by the addition of sodium hydroxide at a concentration of 0.1 mol/L after 8 h in the water bath. The product was centrifuged at 3000 r/min for 10 min and then washed 3–5 times with deionized water. Finally, the product was dried in an oven at 45 °C for 3 h, ground, and prepared.

The adsorption properties of porous starch on SOEO (PS/SOEO microspheres) were analyzed by the differential weight method. The porous starch was stirred at high speed with SOEO and then kept at room temperature to remove the surface residue. The adsorption rate of SOEO was then calculated by the following equation:

Adsorption rate (%) = (m_2_ − m_1_)/m_1_ × 100% (1)

where m_1_ (g) is the weight of the initial porous starch, and m_2_ (g) is the final weight of the porous starch.

### 2.3. Composition Analysis of Sweet Orange Essential Oil (GC–MS)

The sweet orange peel was placed into a thermostatic drying chamber for drying and crushed. Then, 100 g treated sweet orange peel was put into 1000 mL distillation flask with a certain amount of water. The upper oil liquid was sweet orange oil (SOEO) after steam distillation.

The essential oil of sweet orange was analyzed by 7890A-5975C gas chromatography–mass spectrometry (GC–MS). First, 10 μL of SOEO was diluted 1000-fold with n-hexane, and 1.5 μL of diluted solution was placed into the HS-SPME sample flask. The chromatographic conditions were as follows: DB-WAX model chromatographic column (30 × 0.25 mm); inlet temperature of 240 °C; pressure of 100 kPa; and high-purity helium as carrier gas. The initial temperature of 60 °C was held for 4 min, then ramped up to 240 °C at 4 °C/min and held for 2 min. The mass spectrometry conditions were as follows: ion source temperature of 200 °C and interface temperature of 220 °C. The solvent delay time was 5 min [30].

### 2.4. Determination of the Antibacterial Properties of Sweet Orange Essential Oil

Referring to the relevant literature, the Oxford cup method was used to determine the diameter of the essential oil inhibition zone (IZ) [31]. First, 100 μL each of the already cultured *Aeromonas sobria* and *Shwanella putrefaciens* were applied to a suitable solid medium by the spread plate method. Three Oxford cups were placed on the surface of the plates, and 200 μL of SOEO was added to the Oxford cups. An equal volume of sterile water was used as control. After about 24 h, a vernier caliper was used to measure the diameter of the IZ.

### 2.5. Preparation of Sweet Orange Essential Oil Microcapsules

According to the method of Fu et al. [27], 1 g of PS/SOEO microspheres was weighed and 3 g of SA was added and stirred at high speed for 1 h, followed by drop-by-drop addition of 1 g of 10KGY irradiated modified CMCS; the mass ratio of the three materials was 1:3:1. The resulting emulsion was dripped into calcium chloride solution using a syringe and cured in a refrigerator for 2 h. The residue was then dried in an oven to obtain CMCS-SA-PS/SOEO microcapsules. The rates of CMCS and SA are shown in Table 2.

### 2.6. Characterisation of Materials

#### 2.6.1. Morphology of Samples by Scanning Electron Microscopy Analysis (SEM)

The surface morphology of modified starch, natural starch, and SOEO microcapsules was studied by SEM (Hitachi SU8010). The images obtained were 50, 20, and 1 μm.

#### 2.6.2. Fourier Infrared Spectroscopy (FTIR)

Fourier infrared spectroscopy (FTIR, NICOLE 6700) was used to analyze the chemical composition of the samples (modified starch, natural starch, and SOEO microcapsules). First, 1–2 mg of powder sample and 200 mg of pure KBr were finely ground, homogenized, and pressed into a transparent sheet on an oil press. The samples were then put into an infrared spectrometer for testing. The wavenumber range was 4000–400 cm^−1^, and 32 scans at 4 cm^−1^ resolution were achieved for each spectrum.

#### 2.6.3. X-ray Diffraction Analysis (XRD)

To study the packing characteristics and crystallinity of the material, the diffraction intensity of the samples (modified starch, natural starch, and SOEO microcapsules) were recorded every 5° by XRD (Bruker D8 Advance 25) over a 2θ range of 5° to 60° [31]. The results were analyzed by Jade program (9.0) and Origin (9.0).

#### 2.6.4. Pressure Resistance Analysis

The pressure resistance of SOEO microcapsules (Groups A, B, C, and D) was analyzed by a universal mechanical testing machine (CMT6103). The range of the load sensor was 10 kN, and the test speed was 1.5 mm/min. The compression deformation was obtained by displacement of the crosshead of the testing machine, so the compression elastic modulus calculated in this study is the apparent elastic modulus. Each sample was conducted in triplicate.

### 2.7. Study of the Release Kinetics of SOEO Microcapsules

Ethanol is soluble in any proportion with water. It reduces the interfacial tension between EO and water, accelerates the dissolution of EO on the surface of microcapsules, accelerates the release of the core material, and shortens the period of the experiment [18]. For this reason, 50% ethanol was used as a solvent for the release kinetics of the microcapsules. Referring to the method of Liu et al. [32], SOEO was diluted using 50% anhydrous ethanol, and the absorbance of SOEO at a wavelength of 671 nm was tested using a UV spectrophotometer [33]. The linear standard equation was y = 0.1203x − 0.0078, R^2^ = 0.9941.

Next, 0.2 g of SOEO microcapsules was added to 30 mL of 50% ethanol solution in a closed system at room temperature (25 °C). At each time point (5, 10, 15, 45, 55, 75, 100, 150, 180, and 300 min), an amount of the solution was removed and the absorbance at 670 nm (Figure S1) was measured using a UV spectrophotometer.

### 2.8. Determination of the Antibacterial Potential of SOEO Microcapsules

According to the method of Tu et al. [33], 100 μL of *Shewanella putrefaciens* and *Aeromonas sobria* were spread on the medium, and microcapsules of SOEO (0.2, 0.4, 0.6, 0.8, and 1.0 g) were fixed with double adhesive on the lids of the dishes. The culture dishes were then inverted and incubated in a constant temperature (37 °C) incubator for 48 h. The optimum amount of microcapsules was selected by the spread plate count method. The antibacterial properties against composite strains were also examined, and the proportion of composite strains was as follows: *Aeromonas sobria*/*Shwanella putrefaciens* ratio = 1:1, 50 μL.

### 2.9. Packaging of Crawfish with SOEO Microcapsules

Alive crawfish with an average weight of 30 ± 2.4 g and average length of 20 ± 1.8 cm was purchased in June from the Qiyimeng fresh market, Wuhan. Upon arrival to the laboratory, crawfish was cleaned in water, the head and sand vein were removed, and the tail was rinsed. The crawfish was cooked at 100 °C for 7 min and then vacuum packed quickly with SOEO microcapsules (1/10 g) before storing at room temperature (25 °C) in a biochemical incubator (BPN-40CRH, blue pard, Shanghai Bluepard Instruments Co., Ltd., Shanghai, China).

#### 2.9.1. Determination of the Total Volatile Base Nitrogen (TVB-N)

TVB-N value was estimated by the FOSS method. Crawfish was hacked through the packing, cleaned and drained in sterile environment (5.0 g), and homogenized with MgO (0.5 g) using a mixer. Finally, the mixture was placed into a FOSS digestion tube. TVB-N value was measured by an automatic Kjeldahl apparatus (8400 Kjeltec Distillation, FOSS Analytical Instrument Co. Ltd., Shanghai, China), and the level of TVB-N was expressed as mg N/100 g of crawfish.

#### 2.9.2. Determination of the Total Plate Count (TPC)

The TPC value was estimated as follows. First, the crawfish was hacked through the packing, cleaned and drained in sterile environment (10 g), and homogenized. Then, it was placed into a sterile bag with 90 mL aseptic sterile saline and beaten using a beating homogenizer for 3 min to obtain uniform bacterial suspension. The bacterial suspension was diluted by 10× dilution method. The appropriate bacterial solution (0.1 mL) with three gradients was coated on the surface of the plate. The TPC of the dilution was measured using the standard plate count method using nutrient agar after incubating at 30 °C for 48 h, and the value of TPC was expressed as log CFU/g of crawfish.

### 2.10. Statistical Analysis

One-way analysis of variance (ANOVA) was utilized for a completely randomized statistical analysis; *p* < 0.05 was considered statistically significant. The SPSS software (version 26.0) was used for all statistical analyses.

## 3. Results and Discussion

### 3.1. Modified Starch Adsorption Performance Analysis

Table 3 shows the results of orthogonal optimization experiments for the modified starches. The optimum conditions for preparation of porous modified starch were as follows: enzyme/starch ratio of 0.020:1, buffer solution pH of 5.5, and 45 °C water bath temperature. The adsorption of SOEO by the modified starch could reach 17.2%. The impact of the factors was pH > temperature > enzyme/starch ratio. This may be because the pH is the main factor in enzyme activity.

### 3.2. Composition Analysis of SOEO

The chromatograms (shown in Figure S2 and Table 4) revealed the main compounds of SOEO as follows: β-myrcene (35.94%), n-octanal (31.91%), d-limonene (15.38%), 3-carene (3.83%), and pentadecane (1.59%). The total proportion of the five ingredients reached 88.65%, with a very small proportion of other ingredients (<2%).

Other researchers have proven that aldehydes (33.93%) [14] and d-limonene (15.38%) [13] are the main antibacterial agents among these compounds. It has been shown that aldehydes cause bacterial death by inhibiting the activity of enzymes in the cell membrane and causing extravasation of intracellular material [19]. Limonene preserves food by disrupting bacterial cell membranes and cell proteins [20].

### 3.3. Antibacterial Properties of SOEO

**Figure 1:**
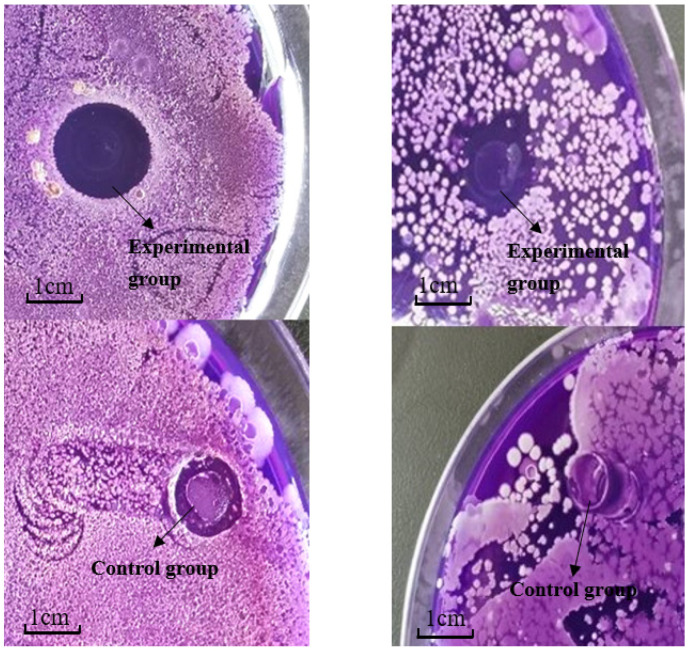
Size of the inhibition zone of the two strains.

In this study, we used *Shewanella putrefaciens* and *Aeromonas sobria* to carry out the bacteriostasis test because our previous studies found these bacteria to be the dominant spoilage bacteria for crawfish stored at room temperature (unpublished data). As shown in Figure 1, obvious inhibition circles were observed in the experimental groups of *Shewanella putrefaciens* and *Aeromonas sobria*. Measurements showed that the inhibition zone size was 1.1 ± 0.1 cm for *Shewanella putrefaciens* and 0.9 ± 0.1 cm for *Aeromonas sobria*. No inhibition zone was observed in the control group of both strains, indicating that SOEO has relatively high inhibition potential against *Shewanella putrefaciens* and *Aeromonas sobria*. Regarding the mechanism of inhibition, He et al. studied the inhibitory effect of eucalyptus essential oil on four strains of bacteria, including *Escherichia coli* and *Shewanella putrefaciens*, and showed that the use of eucalyptus essential oil resulted in increased permeability of bacterial cell membranes and impaired cellular integrity and cellular physiological functions, demonstrating the inhibitory effect of plant essential oil on specific spoilage bacteria in aquatic products [21].

### 3.4. Preparation of SOEO Microcapsules

The modified starch was used as an adsorbent to adsorb SOEO, and SA and CMCS were used as wall materials to encapsulate the modified starch with adsorbed SOEO to form a microcapsule structure. The carboxyl group (−COOH) of SA and the amino group (−NH_2_) of CMCS wrapped the starch between them due to electrostatic attraction to form an electron layer, and white emulsion-like CMCS-SA-PS/SOEO was finally obtained [21]. The SOEO microcapsules were cured in 1.5% calcium chloride solution, and the hardness index became high.

As shown in Table 2, different ratios of CMCS-SA-PS/SOEO microcapsules were prepared by using different mass fractions of SA and CMCS solutions. Microcapsules with the best ratios were selected according to some characterization analysis.

### 3.5. Characterisation of SOEO Microcapsule

#### 3.5.1. SEM Analysis

**Figure 2:**
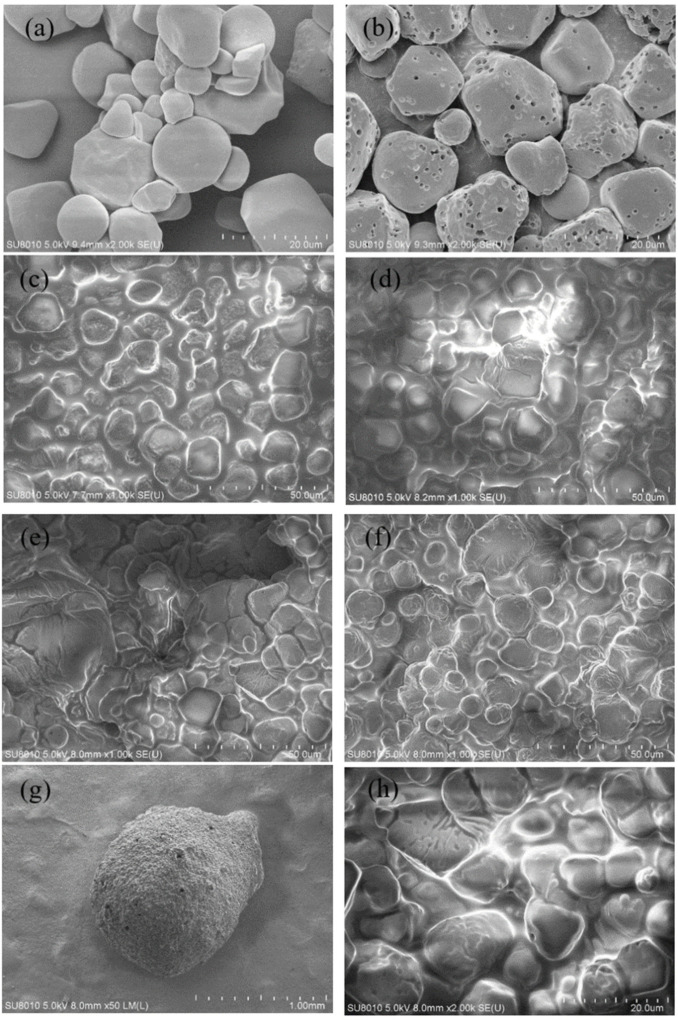
Scanning electron microscopy of starch and microcapsules: (**a**) natural starch; (**b**) modified starch; (**c**) group A microcapsules, magnification ratio: 1000×; (**d**) group B microcapsules, magnification ratio: 1000×; (**e**) group C microcapsules, magnification ratio: 1000×; (**f**) group D microcapsules, magnification ratio: 1000×; (**g**) group C microcapsules, magnification ratio: 50×; and (**h**) group C microcapsules, magnification ratio: 2000×.

The morphology of the natural starch and modified porous starch were observed under 2000× magnification (Figure 2a,b). The natural starch (Figure 2a) was an irregular granule with a smooth surface. The modified starch (Figure 2b) had not significantly changed in shape but had an irregular appearance overall. Moreover, the surface had become rough, and a large number of microporous structures appeared (Figure 2g and Figure S3). This is very similar to the modified starch prepared using enzymatic digestion by other results. Both had many microporous structures on their surfaces [31].

Figure 2c–f shows the A, B, C, and D microcapsules (Table 2) obtained from different material ratios and observed under 1000× magnification. As can be seen, the surface space of microcapsules in group C was smaller than that in the other three groups, proving that group C microcapsules were more tightly wrapped around SOEO. Figure 2g shows the surface morphology of group C microcapsules as observed under 50× SEM. As can be seen, an encapsulated structure was formed on the surface of the microcapsules and no oil-like material was visible on the surface. Figure 2h shows the morphology of group C microcapsules observed under 2000× magnification. The oil-like material was faintly visible, further demonstrating the successful encapsulation of SOEO by the microcapsules.

#### 3.5.2. FTIR Analysis

**Figure 3:**
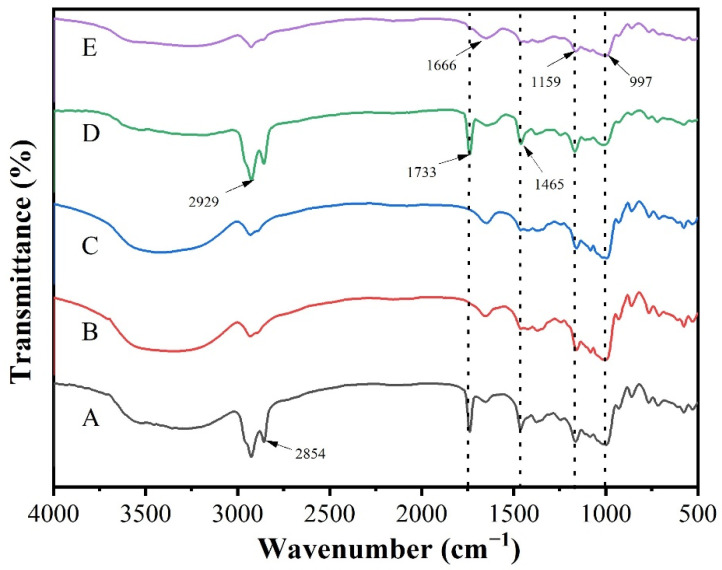
FTIR spectra of (A) PS/SOEO; (B) modified starch; (C) natural starch; (D) SOEO microcapsules; and (E) microcapsules without SOEO.

Figure 3 illustrates the FTIR spectra of modified-starch-adsorbed SOEO, modified starch, natural starch, starch SOEO microcapsules, and microcapsules unloaded with SOEO. The absorption peaks of modified starch (B in Figure 3) and natural starch (C in Figure 3) did not change significantly, indicating that enzymatic reaction did not change the functional groups and the molecular structure of the starch remained essentially the same. The PS/SOEO (A in Figure 3) and SOEO microcapsules (D in Figure 3) had similar characteristic peaks because they both contained SOEO. The characteristic peaks of SOEO were at 1465, 1733, and 2929 cm^−1^. This could be attributed to the presence of alkanes in SOEO with C–H_2_ and C–H_3_, the aldehydes in SOEO with high C–H and C=C stretching intensity and thus a sharper characteristic peak, and O–H stretching, respectively. The peaks at 1159 and 997 cm^−1^ in the microcapsules without SOEO were due to the N–H stretching of the group in CMCS, and the peak at 1666 cm^−1^ was the stretching vibration of the double bond of the carboxyl group in SA [34,35].

#### 3.5.3. XRD Analysis

**Figure 4:**
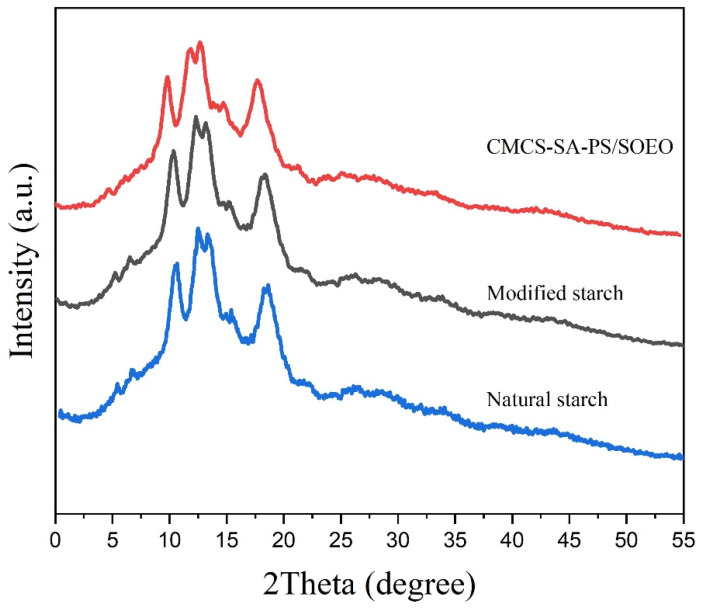
XRD pattern of natural starch; modified starch; and CMCS-SA-PS/EO.

The crystallinity of natural starch, enzymatically modified starch, and SOEO microcapsules are shown in Figure 4. The results showed that the natural maize starch had an A-shaped structure, and the A-pattern was still retained after modification. Previous studies have pointed out that starch granules are amorphous structure as the starch consists of straight-chain starch and twisted or unstable branched chains [36,37]. From the XRD pattern, we can see that the natural (Figure 4, Natural starch) and modified (Figure 4, Modified starch) starches showed strong diffraction peaks at 15° and 23°2θ, double peaks at 17° and 18°2θ, and some smaller diffraction peaks at around 20°2θ Researchers have generally associated imperfections in starch granules with amorphous structural domains, such as broken chains of straight-chain starch and disordered or unstable branched starch. The same properties of starch, such as symmetry, order, and stability, are associated with branched starch [38,39].

In contrast, the peaks in SOEO microcapsules (Figure 4, CMCS-SA-PS/EO) were not very sharp, and the position of the peaks were the same as natural starches. This may be because the compounds of SOEO were very volatile, and its crystallinity was poor.

#### 3.5.4. Pressure Resistance Analysis

**Figure 5:**
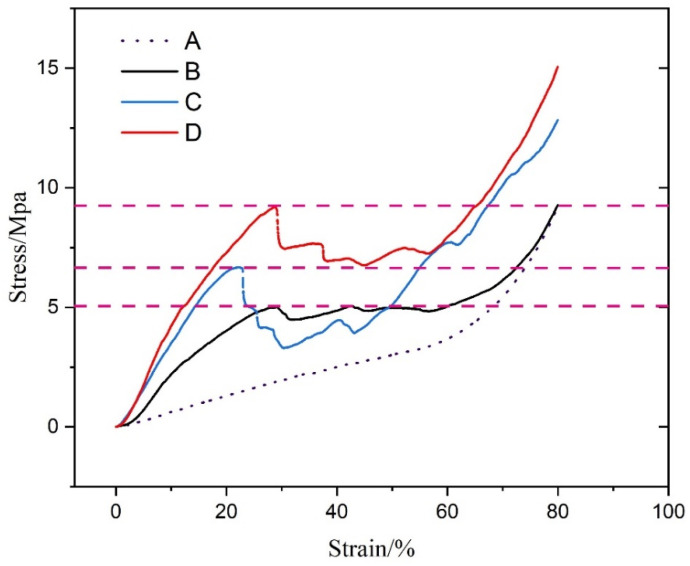
Pressure resistance analysis of (A) group A microcapsules; (B) group B microcapsules; (C) group C microcapsules; and (D) group D microcapsules.

The pressure resistance analysis of SOEO microcapsules is shown in Figure 5. It can be concluded that the higher the rate of CMCS and SA, the higher was the pressure resistance of microcapsules. This might be attributed to the mechanical properties that come from CMCS and SA, which improved the resilient ability of SOEO microcapsules. However, the microcapsules were wrapped with SOEO. The shell would be thicker with increasing rate of CMCS and SA, and a thicker shell is not conducive to the release of SOEO.

### 3.6. Study of the Release Kinetics of SOEO Microcapsules

**Figure 6:**
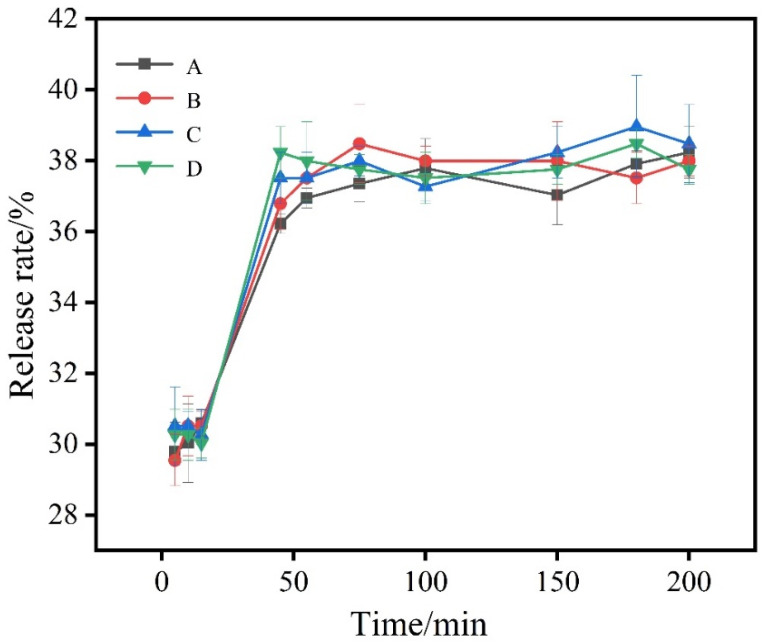
Release curves of (A) group A microcapsules; (B) group B microcapsules; (C) group C microcapsules; and (D) group D microcapsules in 50% ethanol solution.

Figure 6 shows the release of sweet orange essential oil microcapsules in 50% anhydrous ethanol. In the range of 0–200 min, the release curves generally conformed to the mathematical model of y = a × b [40]. After fitting the C capsules by the origin, the resulting release kinetic equation was y = 3.6108 × 1.7226 with a well-fitted correlation coefficient of R^2^ = 0.9716. It can be seen from the graph that SOEO in the four groups was released rapidly in the anhydrous ethanol solution during the period of 0–50 min; after 50 min, the release rate of the four groups of sweet orange essential oil microcapsules tended to stabilize. It can be speculated that some essential oils may have been attached to the surface of the microcapsules at the beginning and when entering the ethanol solution, these surface oils were released into the ethanol solution in a shorter period of time, thus resulting in a faster release rate at the initial stage [28,39].

Although the releasing curves of the four groups of microcapsules were similar, the final release rate of SOEO encapsulated in the microcapsules of group C was about 38%, which was higher than the other groups of microcapsules. On the other hand, it also proved that the best microcapsules were prepared by the material ratio of group C.

### 3.7. Antibacterial Potential of SOEO Microcapsules

The results of the inhibition of *Shewanella putrefaciens*, *Aeromonas sobria,* and the complex bacterium by SOEO microcapsules are given in Table 5. In our experiments, 0.8 g of SOEO microcapsules showed the best antibacterial ability, suggesting that the antibacterial effect of SOEO does not increase with the increase in dose because of its volatility. Compared to natural starch, the ability of inhibition by 0.8 g SOEO microcapsules against *Shewanella putrefaciens*, *Aeromonas sobria*, and the complex bacterium reached 56.43, 61.27, and 62.91%, respectively, at 48 h.

With the extension of time, the antibacterial effect of sweet orange essential oil and SOEO microcapsules was weakened, but SOEO microcapsules showed more persistent antibacterial properties than essential oil on its own. In the initial 12 h, pure essential oil showed excellent antibacterial properties because a large amount of essential oil was released. When the incubation time was extended to 24–48 h, the modified porous starch and chitosan in the microcapsule protected the orange peel essential oil, reduced its instability, and prolonged the antibacterial effect of the essential oil. Moreover, our results showed that the antibacterial effect of microcapsules on *Aeromonas thermophila* was better than that of *Shewanella*. This might be because *Aeromonas thermophila* can use starch substrate, so the EO has better contact with the microbial cell membrane. In the complex flora, microcapsules also showed certain antibacterial ability due to the competitive growth relationship between the two bacteria.

Unlike free essential oils, EO starch microcapsules can be divided into three steps. First, the wall of the microcapsules is diffused (because the compounds of shells are mainly from macromolecule) by degradation and released from the microcapsules; second, the substrate starts to swell and the degradation or corrosion of the shells leads to the release of the EO; third, the released EO works on the bacteria [40]. In this study, we used chitosan to protect essential oil. Compared to other studies that used, for example, the combination of sodium caseinate and baili EO or the combination of sodium alginate and citronella EO, our SOEO microcapsules showed better antibacterial ability because of synergistic bacteriostasis [41].

### 3.8. Research on the Preservation of Crawfish

#### 3.8.1. Total Volatile Base Nitrogen (TVB-N)

**Figure 7:**
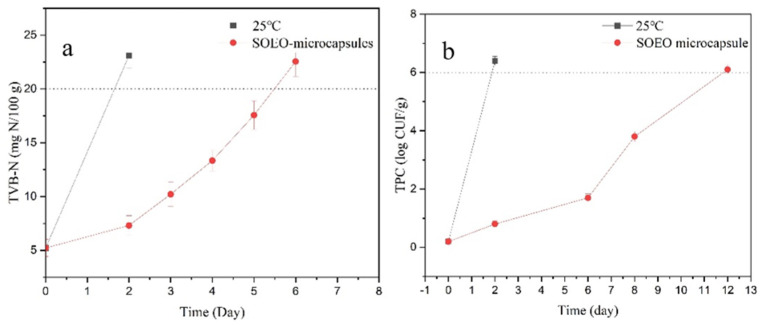
The TVB-N (**a**) and TPC (**b**) of crawfish under 25 °C and SOEO microcapsules.

TVB-N is one of the main indicators used to judge whether aquatic products have decayed. According to hygienic standards for marine products, the acceptable TVB-N values for marine fish and shrimp should be no more than 20 mg N/100 g [42]. As shown in Figure 7a, the TVB-N value of crawfish was 4.21 ± 0.26 mg N/100 g, and it increased with longer time. The TVB-N value of the 25 °C group increased faster and exceeded 20 mg N/100 g on the second day. However, the TVB-N value of SOEO microcapsules exceeded 20 mg N/100 g on the sixth day.

#### 3.8.2. Total Plate Count (TPC)

Figure 7b shows changes in TPC in all crawfish during storage. Crawfish is considered fresh if the TPC is lower than 6 log CFU/g; TPC higher than this means the crawfish is spoilt [42]. On the second day, the TPC value of the 25 °C group was already over 6 log CFU/g. On the sixth day, the TPC value of crawfish stored at 4 °C was 6.17 ± 0.17 log CFU/g.

Overall, SOEO microcapsule showed a good ability in preserving crawfish, and the shelf life of crawfish could be extended to 6 days. Compared to other studies, our SOEO microcapsules were more stable. For example, Alotaibi used sweet potato starch and thyme essential oil to produce microcapsules and found that the rate of lipid oxidation in shrimp meat was significantly inhibited during storage. However, the shelf life of crawfish was only extended to 4 days because the essential oil was not protected in the form of a shell, which led to instability of the essential oil.

## 4. Conclusions

In this study, SOEO microcapsule were prepared by SOEO, modified starch, CMCS, and SA. The modified starch was used as a substance to adsorb SOEO, and the best condition to modify starch was as follows: enzyme/starch ratio of 0.020:1, buffer solution pH of 5.5, and 45 °C water bath temperature. The main antibacterial agents were aldehydes and d-limonene. The results of characterization revealed that (1) SOEO had successfully been adsorbed by modified starch; (2) CMCS and SA could not only wrap the starch well but also improved the pressure resistance of the microcapsule; (3) combined with the results of release kinetics, the best rates of CMCS and SA were 1.2 and 2.4%, respectively. The results of antibacterial test showed that the overall cost-effectiveness of the film was the highest when 0.8 g of SOEO microcapsules was added. In the preservation test of crawfish, it was found that the shelf life of crawfish was greatly extended compared to other treatments.

## Figures and Tables

**Table 1:** Levels and factors of orthogonal experiment.

Serial Number	Influencing Factors		
	Enzyme/Starch Ratio	pH	Temperature (°C)
1	0.015:1	4.5	45
2	0.020:1	5.0	50
3	0.025:1	5.5	55

**Table 2:** Material ratio.

Sample Number	CMCS/%	SA/%
A	0.8	2.4
B	0.8	3.2
C	1.2	2.4
D	1.2	3.2

**Table 3:** Results of the orthogonal experiment.

Serial Number	Influencing Factors			Adsorption Rate (%)
	Enzyme/Starch Ratio	pH	Temperature (°C)	
1	0.015:1	4.5	45	13.07
2	0.015:1	5.0	50	14.28
3	0.015:1	5.5	55	13.39
4	0.020:1	4.5	50	14.28
5	0.020:1	5.0	55	13.69
6	0.020:1	5.5	45	17.22
7	0.025:1	4.5	55	13.94
8	0.025:1	5.0	45	12.60
9	0.025:1	5.5	50	11.36
K_1_	13.58	13.76	14.30	
K_2_	15.06	13.52	13.31	
K_3_	12.63	13.99	13.67	
Signif	0.021	0.02	0.022	
Quadratic sum	124.92	125.58	120.89	

Kn is sum of experimental results at all levels. Signif < 0.05 means this factor has an important influence on the subject. Quadratic sum: the degree of influence of each level on the adsorption.

**Table 4:** Analysis of components of SOEO.

Serial Number	Retention Time	Components	Relative Content (%)
1	23.556	Tridecanal	0.91
2	27.391	D-Limonene	15.38
3	29.675	Octacosane	1.50
4	32.048	Eicosane	1.51
5	32.527	1-Hexanol, 2-ethyl-	0.12
6	32.527	Decanal	0.65
7	35.198	Pentadecane	1.59
8	35.198	Octanal	31.91
9	35.198	Nonanal	0.27
10	35.198	Cyclohexane, 1-ethenyl-1-methyl-2,	0.46
11	35.198	Caryophyllene	0.41
12	35.198	Octadecane	0.48
13	35.198	Dodecanal	0.19
14	35.198	Heneicosane	0.19
15	35.198	Toluene	0.17
16	35.949	β-Myrcene	35.94
17	36.142	β-Pinene	0.68
18	37.604	Naphthalene, 1,2,3,5,6,8a-hexahydr	0.55
19	37.698	Camphene	0.74
20	37.793	3-Carene	3.83

**Table 5:** Bacterial colony count for microencapsulation inhibition test.

Weight/g	CMCS-SA-PS/EO/								
	12 h			24 h			48 h		
	*Shewanella putrefaciens*	*Aeromonas sobria*	Composite Strains	*Shewanella putrefaciens*	*Aeromonas sobria*	Composite Strains	*Shewanella putrefaciens*	*Aeromonas sobria*	Composite Strains
0.2	42 ± 3	35 ± 2	67 ± 2	71 ± 8	113 ± 12	122 ± 10	73 ± 7	137 ± 12	138 ± 10
0.4	38 ± 1	29 ± 3	61 ± 9	70 ± 5	106 ± 13	117 ± 11	72 ± 4	125 ± 13	127 ± 11
0.6	30 ± 3	26 ± 4	63 ± 7	66 ± 3	111 ± 11	109 ± 12	68 ± 4	121 ± 11	119 ± 12
0.8	24 ± 4	11 ± 1	47 ± 3	55 ± 2	103 ± 14	99 ± 8	57 ± 3	106 ± 14	102 ± 8
1.0	27 ± 5	22 ± 5	55 ± 3	61 ± 7	136 ± 11	103 ± 11	64 ± 8	129 ± 11	124 ± 11
CK_2_	20 ± 6	28 ± 8	59 ± 4	81 ± 4	160 ± 15	141 ± 15	89 ± 10	162 ± 15	154 ± 15
CK_1_	61 ± 3	52 ± 4	109 ± 12	99 ± 7	171 ± 18	148 ± 14	101 ± 11	173 ± 18	162 ± 14

CK_2_ represents SOEO with modified starch, and CK_1_ represents the culture condition.

## Data Availability

The data presented in this study are available on request from the corresponding author.

## Supplementary Materials

The following supporting information can be downloaded at: https://www.mdpi.com/article/10.3390/foods11152306/s1, Figure S1: The Full scanning wavelength of SOEO; Figure S2: The chromatograms of SOEO; Figure S3: The Scanning electron microscopy of SOEO microcapsules: a: Group A microcapsules; b: Group B microcapsules; c: Group C microcapsules
