# Supplementary material for: Preparation, Characterization, and Application of Modified Starch/Chitosan/Sweet Orange Oil Microcapsules
Source: Foods. 2022 Aug 2;11(15):2306. doi: 10.3390/foods11152306 (PMC9368646; doi:10.3390/foods11152306)
Supplement: Supplementary file 1 [file foods-11-02306-s001.zip › foods-1830407-supplementary.pdf]

foods
MDPI

Supplementary Materials

Preparation, Characterization, and Application of Modified Starch/Chitosan/Sweet Orange Oil Microcapsules

UH5300

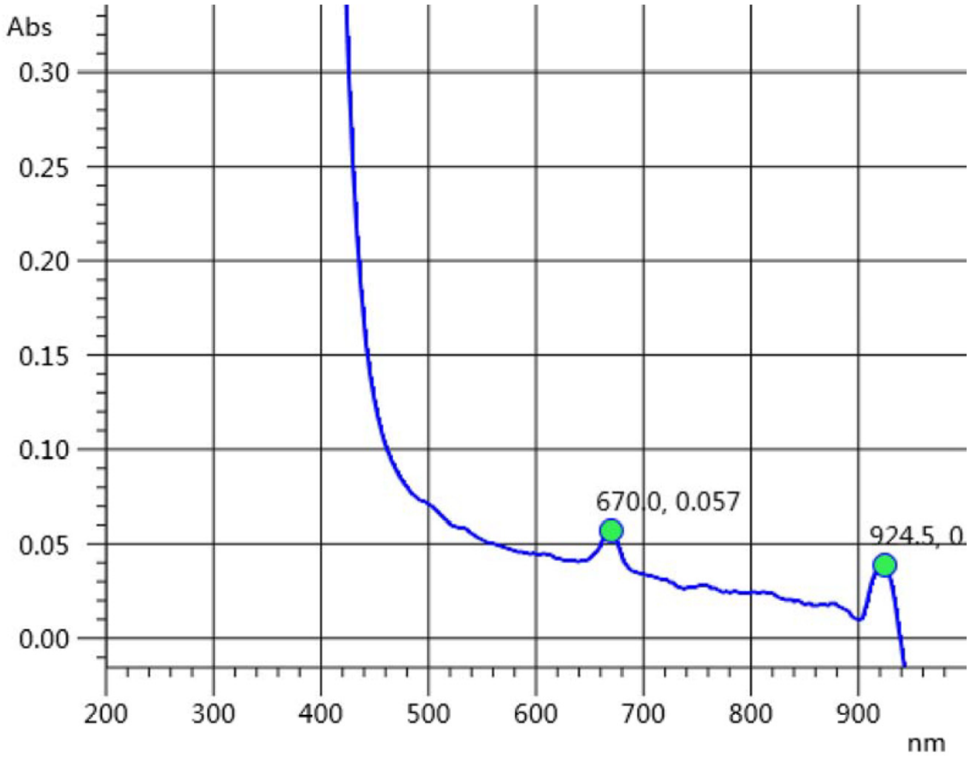

Figure S1. The Full scanning wavelength of SOEO.

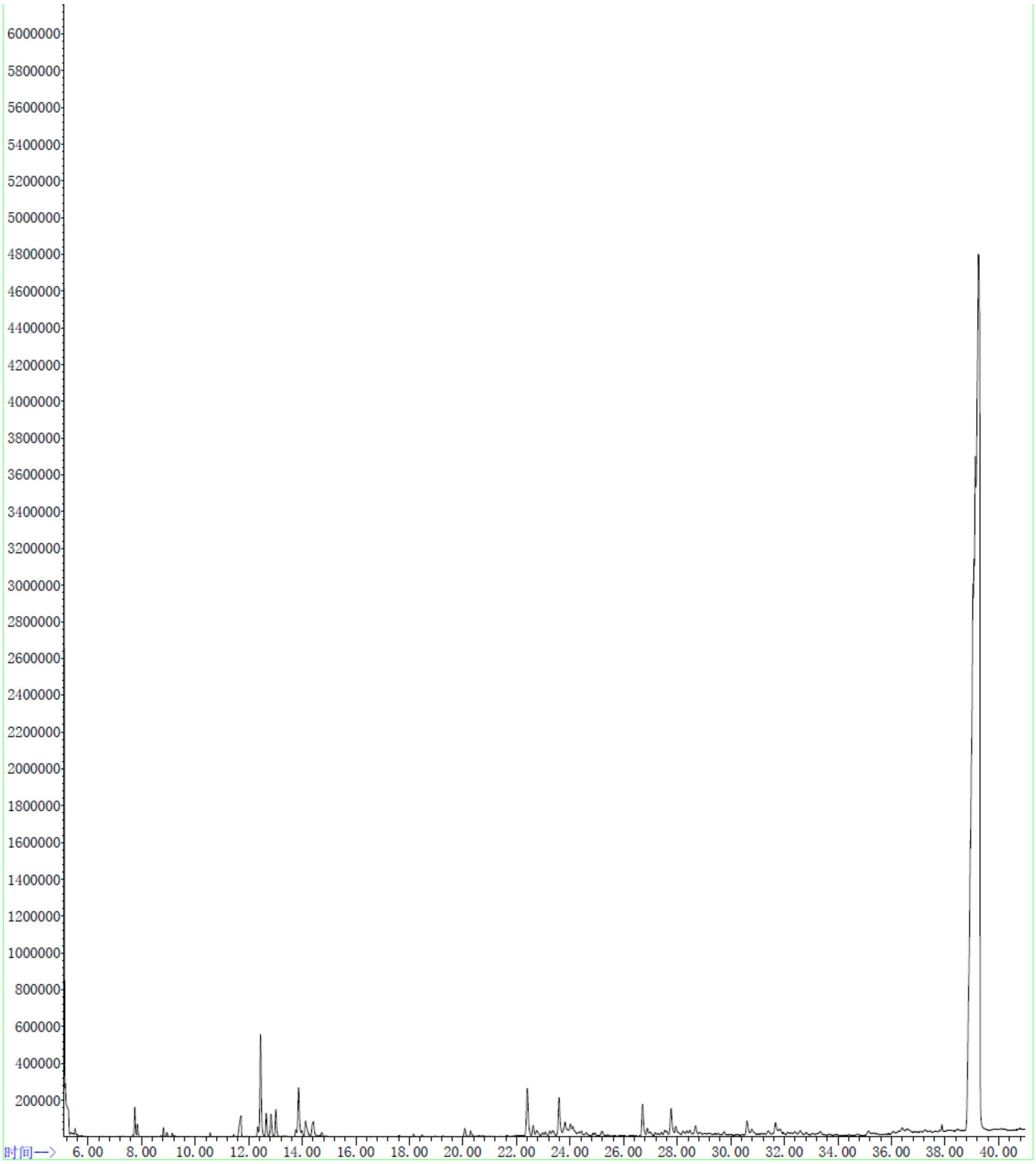

Figure S2. The chromatograms of SOEO

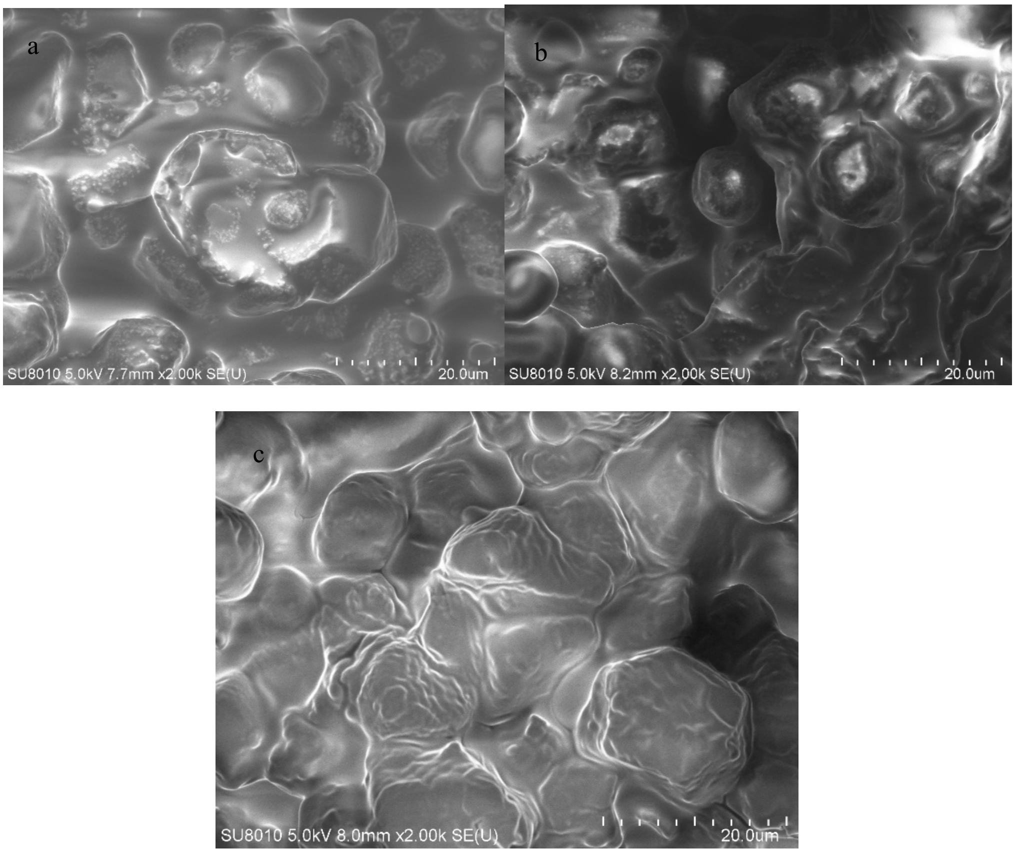

Figure S3. The Scanning electron microscopy of SOEO microcapsules: a: Group A microcapsules; b: Group B microcapsules; c: Group C microcapsules. All authors have read and agreed to the published version of the manuscript.
